# Factors associated with self-rated health in a Norwegian population of older people participating in a preventive home visit program: a cross-sectional study

**DOI:** 10.1186/s12877-020-01733-2

**Published:** 2020-09-04

**Authors:** Astrid Fjell, Seiger Cronfalk Berit, Monica Hermann, Arvid Rongve, Jörg Aßmus, Lars Kvinge, Åke Seiger, Knut Skaug, Anne-Marie Boström

**Affiliations:** 1 Department of Neurobiology, Care Sciences and Society, Division of Nursing, Karolinska Institutet, Stockholm, Sweden; 2 Department of health and caring sciences, Western Norway University of Applied Sciences, Bjørnsonsgate 45, 5528 Haugesund, Norway; 3 Department of Nursing Science, Ersta Sköndal Bräcke University College, Huddinge, Sweden; 4 Department of health and caring sciences, Western Norway University of Applied Sciences, Stord, Norway; 5 Department of Research and Innovation, Helse Fonna, Haugesund Hospital, Haugesund, Norway; 6 Inst. of Clinical Medicine, University of Bergen, Bergen, Norway; 7 Centre for Clinical Research, Haukeland University Hospital, Bergen, Norway; 8 Department of Neurobiology, Care Sciences and Society, Division of Clinical geriatrics, Karolinska Institutet, Stockholm, Sweden; 9 Department of Research and Innovation, Helse Fonna HF, Haugesund, Norway; 10 Theme Aging, Karolinska University Hospital, Huddinge, Sweden

**Keywords:** Self-rated health, Preventive home visits, Older adults, Limited by disease, Information and communication technology, Cross-sectional study design, Linear regression model

## Abstract

**Background:**

Assessing self-rated health by preventive home visits of older people can provide information about the person’s well-being, quality of life and risk of developing illness. The aim of this study was to examine associations between self-rated health and factors related to demographics, lifestyle, health conditions and medical diagnoses by older people participating in a preventive home visit program.

**Methods:**

A cross-sectional study including 233 participants (age 75–79) from three municipalities of Western Norway was conducted. Data were collected through preventive home visits performed by six nurses, using a questionnaire including self-rated health assessment and questions and tests related to demographics (e.g. education and housing), lifestyle (e.g. social activities, alcohol and smoking), health conditions (e.g. sensory impairment, pain and limited by disease) and medical diagnoses. Descriptive and inferential statistics including linear block-wise regression model were applied.

**Results:**

The block-wise regression model showed that the variables Limited by disease and Pain were negatively associated with self-rated health and Use internet was positively associated. The model had a R^2^ 0.432. The variable that contributed to largest change in the model was Limited by disease (R^2^ Change; 0.297, *p*-value< 0.001).

**Conclusions:**

In the present study, being limited by disease and pain were strongly associated with poor self-rated health, indicating that these are important factors to assess during a preventive home visit. Also, digital competence (Use internet) was associated with a better self-rated health, suggesting that it could be useful to ask, inform and motivate for the use of digital tools that may compensate for or improve social support, social contact and access to health -related information.

## Background

This study focuses on self-rated health and preventive home visits (PHV) in older people living in Norway. The worldwide population of people over 60 years is estimated to increase to two billion people in 2050 [1]. It is therefore necessary to focus on the health consequences and challenges with an aging population [2], as aging is associated with increased risk of disease and functional decline, which challenge the health care services to meet new and increasing demands. PHV is a tailored intervention to promote health and prevent illness in older persons, and is considered to be a useful tool to meet challenges with an ageing population [3]. To accomplish optimal effect of PHV, knowledge about the factors associated with the health of older individuals is of vital importance as self-rated health is a good indicator of a person’s general health and conditions.

### Self-rated health

World Health Organization’s definition of health from 1948 “a state of complete physical, mental and social wellbeing and not merely the absence of disease or infirmity” has contributed to develop various health concepts, like self-rated health [4]. Self-rated health is a comprehensive measurement of health status and is broadly used as an indicator to monitor the health of populations and patients in clinical settings [5] and is often used as a screening tool in health surveys or for identifying persons at risk of disease [5, 6]. Even though self-rated health is a wide-ranging measurement, it is based on only one question. The most commonly used question of self-rated health is “in general would you say your health is excellent, very good, good, fair, or poor?” [7]. The response to this modest question is however influenced by many factors, defined by the person being asked. A general assessment of self-rated health can therefore identify aspects of health more accurate than specific questions [5]. By assessing self-rated health, important information about well-being, quality of life, multi-morbidity and predictors of future health are identified. Poor self-rated health has been shown to be an indicator of post-hip fracture mortality and overall early mortality [5, 8, 9].

There are several factors associated with self-rated health, such as the demographic factors gender and education. In a European study with 40,229 individuals aged ≥60, living in 16 countries, 53% of the total sample rated their health as good; 51% of the women and 56% of the men [10]. The tendency of women rating their health poorer than men has also been shown in a longitudinal study in Norway [11] as well as in a prospective study with data from Europe and USA [8]. Studies have shown that level of education is associated with self-rated health and that woman with low educational level or no work outside home rated their heath poorer than women with higher education [8, 12, 13]. There are also differences between countries regarding older persons’ self-rated health. Citizens in Scandinavia and Great Britain rated their health higher compared with citizens in Southern and East European countries [14].

Studies have also shown that lifestyle or social factors are positively associated with self-rated health indicating that they increase the person’s perceptions of good health [10, 12, 15]. Social factors in these studies include variables like social support and participation, household composition, contact with family and attending educational or training courses. Health conditions like number of chronic diseases, low functional status, poor sleep quality and depression are negatively associated with self-rated health, and these associations were found across all countries included in the study [12]. A systematic review showed that poor self-rated health was strongly associated with functional decline by older people living at home [16], and a Norwegian study also identified a need for more health care services [17].

To summarize, self-rated health is a widely used measure for assessing health in older persons. In PHV research assessment of self-rated health is of interest as it provides essential information about the older individual as the rating is based on information that has prognostic power for quality of life, future health and risk of early mortality [18]. The present study is part of a research project where PHVs were used to identify risks among older people [19]. Results from this project showed that poor self-rated health was strongly associated with risk of developing illness, falls, malnutrition and polypharmacy [20]. Despite the wide use of self-rated health measurements and several studies examining associations between self-rated health and various factors, it is still unclear what the self-rated health captures [4, 5, 12, 21]. Due to the central role of self-rated health in age-related challenges and risks, reported in our prior study [20] and other studies [10, 12], we anticipate that new knowledge about factors associated with self-rated health may contribute to increase the efficiency of PHVs. This knowledge could enhance the opportunities to support necessary changes in the older person’s life and thereby improve self-rated health [8]. The aim of this study was therefore to examine associations between self-rated health and factors related to demographics, lifestyle, health conditions and medical diagnoses in older people participating in a PHV program.

## Methods

### Design

This study is part of a larger research project (Health Team for the Elderly) with the aim of identifying risk of developing illness among older people living at home using PHV [19]. A model for PHV was developed using a Comprehensive Geriatric Assessment (CGA) approach. The PHV model was implemented in two municipalities and was found to be feasible [19]. Also, we found that poor self-rated health was associated with risk of developing illness [20]. In this present study a third municipality was included, and a cross-sectional study design was used to examine factors associated with self-rated health.

### Setting and sample

Data were collected from persons of three municipalities, one large urban (45,000 inhabitants), one medium-sized rural (10,000 inhabitants) and one small rural (1000 inhabitants), in the western part of Norway were analysed. A convenience sampling procedure was used. All persons 77 years old in one urban large municipality (*n* = 177) and in one rural medium sized municipality (*n* = 172) were invited to participate in the study. In the medium sized municipality data was collected during a 3 years period and included therefore three cohorts where the participants were 77 years old. The age of 77 years was motivated by studies showed that 75 years old persons seemed to be too young and healthy and those 80+ started to be too old for being suitable for the interventions [22, 23]. Therefore, the decision was made to focus on persons 77 years old [19]. In the main research project, all persons ≥75 in a small rural municipality (*n* = 82) were invited to participate, however in the present analyses only individuals aged 75–79 were included. Inclusion criteria were; living at home, irrespective of whether they received home assistance from the municipality or not. Exclusion criteria were; older persons living in a nursing home and/or unable to communicate in Norwegian language (reading or writing). In this study we also excluded persons older than 79 years to have a more homogeny sample.

### Data collection

Data were collected at PHVs conducted in the three municipalities. Six trained nurses used a questionnaire including validated tests on various risks related to ageing; for example, self-rated health using SF-1 [24], positive life orientation scale (PLOS) [25, 26], social support using OSLO 3-SSS [27] and pain using VAS [28], in addition to questions regarding demographics, lifestyle and health conditions including medical diagnoses. All participants received an invitation letter with information about the study. An administrator phoned each person, and if the older person wanted to participate, an appointment was set up for a PHV. The development of the questionnaire and the data collections procedures has previously been described in detail by Cronfalk and colleagues [19].

Halfway through the study, the questionnaire and data collection procedure were revised, as some questions were sent ahead of the visit rather than being posed during the PHV. These questions concerned; gender, education, marital status, living conditions, have mobile phone and use internet, hobbies, social support and participation in organizations and voluntary work. Also, the questions about “How would you describe your vision?” was replaced with a question about sensory impairment.

### Dependent and independents variables

In this study self-rated health was the dependent variable. It was measured using SF-1 item “How do you rate your health?” with the response options poor/fair/good/very good/excellent health. The response options were dichotomized into good (excellent/very good/good) or poor (fair/poor) health in the descriptive analyses. In the regression model analyses a five-point Likert scale was used (poor health = 1, excellent health = 5).

To assess factors related to demographics, lifestyle, health conditions and medical diagnoses 32 questions from the interview guide were selected. Most independent variables were selected based on earlier literature about factors associated with self-rated health. The medical diagnoses chronic obstructive pulmonary disease (COPD), hearth failure, stroke and cancer were chosen based on that they are the most common medical diagnoses which causes death in the Norwegian population 65 years and older [29]. Anxiety was included because anxiety and depression are often related to suicide among older [30], which is increasing in Norway [29]. Osteoporosis and arthrosis were included due to that these diagnoses are related to pain and reduced movement patterns and can had an influence on a person’s life [31]. However, factors hypothesized to be associated with self-rated health (have mobile phone and use internet), based on clinical experience within the research team and the development of digital tools in Norway, were also included despite lack of previous studies. The independent variables were categorized into four groups for conducting the block-wise regressions analysis; 1. Demographic variables (e.g. age, gender, education, marital status and living conditions), 2. Lifestyle variables (e.g. social support, exercise, external activities, smoking, use alcohol, have mobile phone and use internet) 3. Health condition variables (e.g. vision, hearing, sleep problems, pain, limited by disease, home care service) and 4. Medical diagnoses (e.g. COPD, stroke and arthroses). The dependent and independent variables including response options are presented in Table 1.

**Table 1:** Description of the dependent and independent variables

**Dependent variables**			
**Variable**	**Instrument/question**	**Response options**	**Use in the analysis**
Self-rated health	SF 36. Item 1. How do you rate your health?	Poor health = 1 Fair = 2 Good = 3 Very good = 4 Excellent = 5	Continuous
Self-rated health	SF 36. Item 1. How do you rate your health?	Poor health = 1 Fair = 2 Good = 3 Very good = 4 Excellent = 5	Dichotomized Poor = 0 (fair, poor) Good = 1 (good, very good, excellent)
**Independent variable**			
**Variable**	**Instrument/question**	**Response options**	**Use in the analysis**
**Demographics**			
Age	What is your birth date?	Years	Continuous
Gender	Which gender are you?	Male = 0 Female = 1	Dichotomous
Education	Which education do you have?	≤7 years elementary school Middle school Secondary school High school Folk high school Craftsman education Trade school University Other	Dichotomized No college = 0 (≤7 years, elementary school, middle school, secondary school, high school, folk high school, craftsman education, other) College = 1 (trade school, high school, university)
Municipality	In which municipality you live?	Large municipality =1 Medium municipality = 3 Small village =2	Categorical
Marital status	What is your marital status?	Married Cohabitant Alone Divorced Widow(er)	Dichotomized Single = 0 (alone, divorced, widow/er) Partner = 1 (married, cohabitant)
Living alone	Do you live alone?	Yes = 0 No = 1	Dichotomous
Residential	Is the residential:	Hired = 0 Owned/Private = 1	Dichotomous
Improvements in house	Is it necessary to do improvement in your house?	Yes = 0 No = 1	Dichotomous
**Lifestyle**			
Social support	OSLO 3 – SSS		Continuous
	1. How many people are standing so close that you can count on them if you have big personal problems	No one (1 p) 1 to 2 (2 p) 3 to 5 (3 p) 6 or more (4 p)	
	2. How interested are people for what you do?	No participation or interest (1p) Little participation and interest (2p) Unsure (3 p) Some participation and interest (4 p) Great participation and interest (5 p)	
	3. How easy is it to get practical help from neighbours if you need it?	Very difficult (1p) Difficult (p) Possible (3p) Ease (4p) Very easy (5 p) 3–8 p = Lack of support 9–11 p = Some support 12–14 p = Much support	
Exercise	How often do you exercise? (minimum 30 min duration so that you are warm and slightly out of breath)	Rare = 1 1–3 times a month = 2 1–3 times a week = 3 4–6 times a week = 4 Daily = 5	Continuous
Smoking	Do you smoke?	Yes No No, but smoked earlier	Dichotomized Smoking = 0 No smoking = 1 (no; no, but smoked earlier)
Use alcohol	Do you use alcohol?	Yes = 0 No = 1	Dichotomous
External activities	How often are you away from home?	Rare = 1 1–3 times a week = 2 4–6 times a week = 3 Daily = 4 Several times a day = 5	Continuous
Have mobile phone	Do you have a mobile phone?	No = 0 Yes = 1	Dichotomous
Use Internet	Do you use Internet?	No = 0 Yes = 1	Dichotomous
Have a hobby	Do you have a hobby?	No = 0 Yes = 1	Dichotomous
Participate in a club/social organized activity	How often do you participate in a club/social organized activity?	Never = 1 Seldom = 2 1–3 a month = 3 1–3 a week = 4 4–6 a week = 5 Daily = 6	Continuous
**Health conditions**			
Vision (Two different question used in different questionnaire)	How would you describe you vision? Downton fall risk index (only visions impairment)	Visually impaired Reduced Somewhat impaired Good Excellent If the person has vision impairment, drew a circle around 1.	Dichotomized Reduced = 0 (somewhat impaired, reduced, visually impaired) Good = 1 (excellent and good)
Hearing	How would you describe you hearing?	Hearing impaired = 1 Reduced = 2 Somewhat impaired = 3 Good = 4 Excellent = 5	Continuous
Sleep problems	Do you have sleep problems?	Yes = 0 No = 1	Dichotomous
Pain	Do you have pain?	Yes = 0 No = 1	Dichotomous
Life orientation	a. Are you satisfied with your life? b. Do you feel someone need you? c. Do you have plans for the future? d. Do you have zest for life? e. Do you feel lonely? f. Do you feel depressed/sad	a. - d: Yes/No e. – f: Rarely or never/sometimes/often or always “Yes” rated at questions a – d and “Rarely or never” at questions e and f = Positive life orientation	Dichotomous Negative = 0 Positive = 1
Home care service	Do you have public help at home?	No Home Care Home help Food delivery Drugs or medications	Dichotomized Yes = 0 (home care/home help/food delivery/drugs or medications) No = 1 (no)
Limited by disease	Does the disease affect you in everyday life?	Completely = 1 Very much = 2 Often = 3 Sometimes = 4 Not at all = 5	Continuous
**Medical diagnoses**			
Stroke	Have you had a stroke?	Yes = 0 No = 1	Dichotomous
Heart failure	Do you have heart failure	Yes = 0 No = 1	Dichotomous
COPD	Do you have COPD	Yes = 0 No = 1	Dichotomous
Cancer	Do you have cancer	Yes = 0 No = 1	Dichotomous
Depression	Do you have depression?	Yes = 0 No = 1	Dichotomous
Anxiety	Do you have anxiety?	Yes = 0 No = 1	Dichotomous
Osteoporosis	Do you have osteoporosis?	Yes = 0 No = 1	Dichotomous
Arthrosis	Do you have arthroses?	Yes = 0 No = 1	Dichotomous

Due to the change of question in the questionnaire, response options to the question about vision used the response options “no” or “reduced vision” from the Downton fall risk index. If the person had answered “no” it was plotted the data set as “good” (vision) and it the answer was “reduced vision” it was plotted as “reduced” (vision). Missing data for most questions were few (< 5%). However, 89 persons (38%) were not assessed for vision and 36 persons (15%) were not assessed for being limited by disease.

### Data analysis

Linear block-wise regression analysis was used to explore associations between the dependent variable self-rated health and the independent variables. Since we had too many independent variables to assess all in one model we had to develop a final model with a reduced number of independent variables [32]. To select the variables for the final model we estimated the univariate model for each independent variable and multivariable models including all independent variables within each of the blocks demographic, lifestyle, health conditions and medical diagnoses. All independent variables with a *p*-value less than 0.1 in at least one of the models were included in the final model. Additionally, we selected variables of clinical relevance even if they were insignificant in both models. For the final model we defined new blocks and included them cumulatively in the model in the following order: Limited by disease (single variable), lifestyle, demographics, health conditions and medical diagnoses. For each new block we estimated improvement of explained variance by including it and used an ANOVA to test if this improvement was significant. Multicollinearity between independent variables was tested using Spearman Rank order test (ρ ≤ 0.85) and the normality assumptions were assessed by a Q-Q-plot.

The general significance level was set to 0.05. To take into account multiple testing effects we used the Bonferroni adjustment in the final model leading to a marginal level of 0.0036. The data were analysed using SPSS 25 (IBM Corporation, Armonk, NY, USA).

## Results

### Description of sample

The total sample consisted of 233 participants (62% of the eligible population, 75–79 years). Approximately half of the included individuals were women and the mean age of the total sample was 77 years. One quarters of the total sample had college as educational level and close to a half were living alone. Nearly all owned their house and about a quarter needed to do Improvements in their house in order to remain home-dwelling.

A majority (163, 70%) of the participants rated their health as good using the response options excellent, very good or good, and 70 persons rated their health as fair or poor. The mean value of the self-rated health for the total sample was 2.84 (SD 0.88). While there were no significant differences between the participants with self-rated good and poor health in demographics and medical diagnoses, the participants with good health reported more use of alcohol, internet and mobile and significantly less sleep problems, pain, negative life orientation, use of home care service and being limited by disease (Table 2).

**Table 2:** Characteristics of the sample according to their self-rated health

			Self-rated health				*p*-value
	Total sample		Good		Poor		
	*n* = 233		*n* = 163		*n* = 70		
	Valid N	Value	Valid N	Value	Valid N	Value	
**Demographics**							
Age^a^	232	77.1 (0.7)	162	77.1 (0.7)	70	77.1 (0.8)	0.412
Female^b^	233	122 (52%)	163	84 (51%)	70	38 (54%)	0.700
College^b^	233	58 (25%)	163	43 (26%)	70	15 (21%)	0.423
Municipality^b^	233		163		70		0.984
Large		108 (46%)		75 (46%)		33 (47%)	
Medium		94 (40%)		66 (41%)		28 (40%)	
Small		31 (13%)		22 (14%)		9 (13%)	
Single^b^	232	92 (40%)	162	67 (41%)	70	25 (36%)	0.420
Living alone^b^	232	96 (41%	163	71 (44%)	69	25 (36%)	0.300
Private residential^b^	231	225 (97%)	161	157 (98%)	70	68 (97%)	0.870
Improvement needed in house^b^	223	59 (27%)	155	39 (25%)	68	20 (29%)	0.508
**Lifestyle**							
Social Support^a^	229	7.8 (2.7)	160	7.7 (2.9)	69	8.0 (2.4)	0.493
Exercise^a^	230	2.8 (1.4)	161	2.8 (1.4)	69	2.6 (1.5)	0.278
Smoking^b^	224	32 (14%)	155	22 (14%)	69	10 (15%)	0.953
Use alcohol^b^	224	125 (56%)	125	95 (61%)	69	30 (44%)	0.013
External activities^a^	232	3.7 (1.0)	162	3.8 (1.1)	70	3.7 (1.0)	0.699
Have mobile phone^b^	233	207 (89%)	163	151 (93%)	70	56 (29%)	0.005
Use Internet^b^	232	102 (44%)	162	82 (51%)	70	20 (29%)	0.002
Have a hobby^b^	232	214 (92%)	162	152 (94%)	70	62 (89%)	0.170
Participate in a club^a^	230	1.4 (1.3)	160	1.3 (1.3)	70	1.4 (1.3)	0.672
**Health conditions**							
Good vision^b^	145	114 (79%)	101	79 (78%)	44	35 (80%)	0.858
Hearing^a^	227	3.9 (0.7)	159	3.9 (0.7)	68	3.8 (0.7)	0.174
Sleep problems^b^	225	65 (29%)	159	36 (23%)	66	29 (44%)	< 0.001
Pain^b^	223	100 (45%)	153	54 (35%)	70	46 (66%)	< 0.001
Negative life orientation^b^	233	92 (40%)	163	52 (31.9)	70	40 (57%)	< 0.001
Home care service^b^	232	41 (18%)	163	21 (13%)	69	20 (29%)	0.003
Limited by disease^a^	197	4.2 (1.0)	138	4.6 (0.7)	59	3.3 (1.2)	< 0.001
**Medical diagnoses**							
Stroke^c^	232	17 (7%)	162	13 (8%)	70	4 (6%)	0.376
Heart failure^c^	232	19 (8%)	162	11 (7%)	70	8 (11%)	0.177
COPD^c^	232	9 (4%)	162	5 (3%)	70	4 (7%)	0.271
Cancer^c^	232	35 (15%)	162	26 (14%)	70	9 (13%)	0.342
Depression^c^	233	4 (2%)	163	1 (1%)	70	3 (4%)	0.082
Anxiety^c^	233	3 (1%)	163	1 (1%)	70	2 (3%)	0.215
Osteoporosis^c^	233	19 (12%)	163	14 (9%)	70	5 (7%)	0.469
Arthrosis^c^	233	32 (14%)	163	20 (12%)	70	12 (17%)	0.215

^a^Mean (SD), t-test, ^b^N(%), χ^2^-test ^c^N(%) Fisher’s Exact Test

### Factors associated with self-reported health

After the univariate and block-wise full model analyses, Education, Use alcohol, Have mobile phone, Use internet, Have a hobby, Hearing, Sleep problems, Pain, Negative life orientation, Home care service, Limited by disease and Depression remained in the final model. Gender and Social support were added to the final model because of clinical relevance (Table 3).

**Table 3:** Univariate analysis of self-rated health (dependent variable) and factors related to demographics, lifestyle, health conditions and medical diagnoses (independent variables) for selection to block-wise regression

	Univariate models		Block-wise full models		Selected
	B (95%CI)	*p*-value	B (95%CI)	*p*-value	variables
**Block 1: Demographics**					
Age	−0.08 (− 0.23, 0.08)	0.317	− 0.07 (− 0.24, 0.10)	0.436	
Gender	−0.04 (− 0.27, 0.18)	0.696	− 0.06 (− 0.32, 0.20)	0.660	x
Education	0.23 (− 0.03, 0.50)	0.078	0.25 (−,0.28, 0.53)	0.078	x
Municipality	0.06 (− 0.06, 0.19)	0.306	0.05 (− 0.09, 0.18)	0.501	
Marital status	0.06 (−0.17, 0.29)	0.613	0.18 (−0.37, 0.73)	0.515	
Living alone	0.00 (−0.23, 0.23)	0.988	−0.21 (− 0.75, 0.32)	0.428	
Residential	0.35 (−0.37, 1.07)	0.340	0.23 (−0.60, 1.05)	0.585	
Improvements in house	0.12 (−0.15, 0.38)	0.393	0.06 (−0.22, 0.34)	0.669	
**Block 2: Lifestyle**					
Social support	0.01 (−0.03, 0.06)	0.518	0.00 (− 0.04, 0.05)	0.898	x
Exercise	0.03 (−0.05, 0.11)	0.505	0.01 (−0.08, 0.10)	0.814	
Smoking	0.25 (− 0.07, 0.59)	0.143	0.16 (−0.19, 0.50)	0.367	
Use alcohol	−0.26 (− 0.49, − 0.02)	0.034	−0.22 (− 0.47, 0.03)	0.090	x
External activities	0.01 (−0.01, 0.03)	0.175	−0.02 (− 0.14, 0.10)	0.784	
Have mobile phone	0.56 (0.20, 0.91)	0.002	0.37 (−0.01, 0.75)	0.057	x
Use Internet	0.46 (0.24, 0.68)	< 0.001	0.38 (0.13, 0.63)	0.003	x
Have a hobby	0.49 (0.07, 0.91)	0.023	0.50 (0.04, 0.96)	0.035	x
Participate in a club	−0.05 (− 0.14, 0.04)	0.308	−0.05 (− 0.14, 0.04)	0.304	
**Block 3: Health conditions**					
Vision	0.15 (−0.01, 0.31)	0.692	0.19 (−0.08, 0.47)	0.167	
Hearing	0.22(−0.03, 0.46)	0.085	0.09 (− 0.10, 0.27)	0.354	x
Sleep problems	0.47 (0.23, 0.72)	< 0.001	0.25 (−0.02, 0.51)	0.071	x
Pain	0.49 (0.26, 0.72)	< 0.001	0.12 (−0.12, 0.36)	0.321	x
Life orientation	0.40 (0.18, 0.63)	< 0.001	−0.03 (− 0.29, 0.22)	0.789	x
Home care service	0.50 (0.21, 0.79)	< 0.001	0.24 (−0.07, 0.55)	0.130	x
Limited by disease	0.46 (0.36, 0.56)	< 0.000	0.46 (0.33, 0.59)	< 0.001	x
**Block 4: Medical diagnoses**					
Stroke	0.11 (−0.33, 0.55)	0.625	−0.04 (− 0.48, 0.40)	0.854	
Heart failure	−0.34(− 0.76, 0.07)	0.105	0.31 (− 0.12, 0.73)	0.155	
COPD	−0.41 (−1.00, 0.18)	0.169	0.38 (−0.21, 0.98)	0.202	
Cancer	0.17 (−0.37, 0.27)	0.768	0.06 (−0.26, 0.38)	0.727	
Depression	−0.86 (− 1.72, 0. 01)	0.053	0.86 (− 0.06, 1.77)	0.067	x
Anxiety	−0.51 (−1.52, 0.49)	0.315	0.12 (−0.95, 1.18)	0.830	
Osteoporosis	−0.11(− 0.53, 0.30)	0.590	0.09 (− 0.35, 0.52)	0.694	
Arthroses	−0.25 (− 0.58, 0.08)	0.134	0.22 (− 0.13, 0.56)	0.211	

^1^Selected because of clinical relevance. ^2^*p* < 0.1

In the final model, only Limited by disease showed a significant association with Self-rated health, B(CI) = 0.37 (0.26, 0.48), *p* < 0.001, and explained alone 30% of its variance, R^2^ = 0.30. Both the lifestyle block, R^2^ change 0.07, *p* = 0.003, and the health conditions block, R^2^ change 0.05, *p* = 0.012, contributed weakly but significantly to the model quality, while demographics and medical diagnoses did not. Even if not significant with respect to the marginal Bonferroni level, we observed within the two contributing blocks Use internet, B(CI) = 0.25 (0.02, 0.47),*p* = 0.033, and Have a mobile phone, B(CI) = 0.32 (− 0.01, 0.65), *p* = 0.054 as well as Pain B(CI) = 0.25 (0.03, 0.47), *p* = 0.023 with a coefficient in a similar range of Limited by disease and a low *p*-value (Table 4).

**Table 4:** The model summary of the block-wise regression analysis

	Final model^a^		Model properties^b^		
			R^2^		
	B (95%CI)	*p*-value	value	change	*p*-value^c^
**Block 1: Single variable**			0.30	–	< 0.001
Limited by disease	0.37 (0.26, 0.48)	< 0.001			
**Block 2: Lifestyle**			0.37	0.07	0.003
Social support	−0.00 (−0.04, 0.04)	0.835			
Use alcohol	−0.11 (0.32, 0.11)	0.332			
Have mobile phone	0.32 (−0.01, 0.65)	0.054			
Use internet	0.25 (0.02, 0.47)	0.033			
Have a hobby	−0.06 (−0.47, 0.35)	0.776			
**Block 3: Demographics**			0.37	0.00	0.354
Gender	0.02 (−0.21, 0.24)	0.891			
Education	0.12 (−0.13, 0.37)	0.334			
**Block 4: Health conditions**					
Hearing	0.03 (0.11, 0.18)	0.651	0.42	0.05	0.012
Sleep problems	0.17 (−0.06, 0.40)	0.150			
Life orientation	0.18 (0.04, 0.39)	0.100			
Pain	0.25 (0.03, 0.47)	0.023			
Home care services	0.21 (−0.06, 0.49)	0.125			
**Block 5: Medical diagnoses**			0.43	0.01	0.108
Depression	0.64 (−0.14, 1.43)	0.108			

^a^Estimation of the model containing all variables in the table. ^b^R-square for the cumulative model and change of R-square by adding the current block. ^c^ANOVA

## Discussion

The aim of this study was to examine associations between Self-rated health and factors related to demographics, lifestyle, health conditions and medical diagnoses in older people who participated in a PHV program. The main findings were that, the variables Limited by disease and Pain were associated with poor self-rated health and the variable Use internet was associated with good self-rated health in the regression model. In the model summary the variable Limited by disease contributed to the highest change (R^2^ change 0.300). In the following we will discuss the associated factors and their implications for improving the PHV model and increase the efficiency of PHV.

Limited by disease and Pain were significantly associated with poor self-rated health and this finding is in line with previous studies. Pain is found to be associated with poor self-rated health among older people living at home [33] and is also associated with being a limitation negatively affecting the everyday life among older persons living at home [34]. The variable being Limited by disease captures not only functional limitation, but also the person’s perceptions of being restricted and affected in daily life from other perspectives than physical function. A qualitative study among frail older persons living at home in Sweden highlight this. The study showed that the older persons felt restricted by their diseases because their choices of activities were reduced and they felt lonely and isolated [35]. Other studies have reported that not being able to do leisure- and everyday activities was more strongly associated with poor self-rated health than medical diagnoses and chronic diseases [13, 36].

WHO recommends that interventions directed towards older people should support self-care management and that individual needs are assessed with the goal of maintaining functional ability and healthy life [37, 38].The negative association between the variables Limited by disease and Self-reported health indicates that there is a need to support older people in managing their lives to minimize restrictions imposed by diseases they experience daily life. The purpose would be to strengthen the possibility for the person to participate in the community, social and other activities of value for them. Nivestam and colleagues (2020) emphasized the importance of a dialogue about challenges and limitations in everyday life that will give the older person a voice to reflect over their own assets and abilities to improve their health situation [39].

In the present study, the variable Use internet was positively associated with self-rated health. Have a mobile phone also showed a tendency to have a positive association with self-rated health. Use of Information and Communication Technology (ICT) was also positively associated with good self-rated health in a study among older people living at home in Sweden [39]. To use mobile phone and internet have been identified to be important tools for older persons to get in contact with others and to conduct health-related tasks, which was related with better self-rated health [40]. Another explanation to the positive association between use of ICT and self-rated health in our study could be that persons using internet are healthier and have a higher education than non-users [41]. Nevertheless, the positive association between self-rated health and the variable Use internet is important to consider for the development of a PHV program, intended to strengthen the older person’s potential and ability to use ICT. In health care services, digitalization is used to inform and communicate with older persons with the purpose to contribute to more effective and improved services [42, 43]. A challenge, however, with using digitalization in health care services is digital exclusion of older people [44].

An unexpected result in our study was that social support as measured by OSLO 3-SSS was not significantly associated with self-rated health. A possible explanation could be that the group in our study used substitutes for social support. More important than social support is how social relationships contribute to intimacy and confidence. Such relationships could belong to neighbourhood groups, religious groups or non-governmental organizations [45]. Many of the participants in our study had a hobby (92%) and often attended external activities (83%) that may include interactions with other persons and therefore represent a substitute for social support. This is supported by the findings by Machón and colleagues (2016), who found that social group activities like hobbies and social events, but not social support, were associated with self-rated health [12].

To summarize, the most important findings for improving the PHV program were the two variables Limited by disease and Use Internet association with Self-rated health. Many frail older persons living at home have high symptom burden like pain, dry mouth, numbness/tingling in hands/feet and lack of energy which will influence their self-rated health [35]. This indicates that health care professionals and suggested interventions should focus on a function-centred view and not only on a disease perspective [36]. Due to that self-rated health is based on subjective and individual judgement, the implication is that during the PHV it is necessary to encourage the older person to describe what he or she thinks and feels are the limitations in life related to health and why [21, 46]. It could be advantageous to let a person explain how they understand their situation, discuss and teach-back advises and views which are considered is an effective method to improve a person’s knowledge and skills regarding their health situation, [47], and thereby increase the self-management of these conditions and health of the older persons. This kind of encouragement can also, in addition to improve the older person’s self-rated health, prevent or delay pre-frailty or frailty [48]. Furthermore, it is central to ask about self-rated health and what he or she includes in the rating of health. Older persons often focus more on how they can maintain everyday life or what hinders them to do what they want, and less on medical diagnoses [49]. Due to the positive association between the variable Use internet with self-rated health it is important to support older people to become familiar with ICT. In relation to good self-rated health, poor ICT skills could be a threat to older persons as they have reduced ability to communicate or interact with other persons or reduced access to health care services [44]. The fast technological expansion during the last decades has opened new possibilities for use of ICT in health care, including care for older people living at home. But new technology also creates new critical factors for user satisfaction [50]. Due to that, training programs for older persons on how to handle ICT and information are needed and recommended to improve ICT skills [51].

A major strength of this study was the use of a questionnaire including validated instruments assessing health and lifestyle factors. The development of the questionnaire and training of the nurses was found to be feasible and reliable and is reported in a previous study [19]. The study was conducted in three non-neighbouring municipalities in Western Norway, both urban and rural and from two different counties. The response rate was approximately 62%, which is considered satisfactory for this type of study [52]. Overall, there were few missing data in the data set but there exist some missing items for some variables, perhaps due to variation among the data collectors over time. All data were self-reported, and we did not check the correctness of the information with the person’s patient record or the person’s general practitioner. Our findings about the proportion of older persons who reported good health (70%) are in line with previous findings of 63 to 76% from a range of European countries for this age group [14, 17]. The assessment of self-rated health using a one-item measurement has strong evidence to be a key factor of the health-related quality of life [4]. Self-rated health is also suitable as an individual assessment, not only as a measure of population health [53]. Apart from the practical aspects of a one-item assessment it must be noted that a subjective one-item assessment is also subjected to weaknesses as it does not give the opportunity to collect information about what is included in the rating. The main limitation of this study is the use of a cross-sectional study design, which means that cause-effect relationships could not be established. Even so, our study contributes to increased knowledge on factors associated with self-rated health, as called for by several researchers [4, 12] and our findings of ICT use is of particular relevance for future studies within this field.

## Conclusions

In this study, being limited by disease and pain were identified to be associated with poor self-rated health. The negative association between limited by disease and self-reported health indicate that it could be useful during a PHV to focus on how to support older people in managing their lives in order to minimize restriction by disease. Also, we observed a positive association between use of internet and self-rated health. This finding indicate that it could be important to motivate for the use internet as it may both compensate for or improve social support and improve access to health-related information.

## Data Availability

The datasets generated and analysed during the current study are not publicly available due to lack of permission from the Ethical review board but are available from the corresponding author on reasonable request.

## Abbreviations

CGA: Comprehensive geriatric assessment
PHV: Preventive home visits
