# Correction to: Factors associated with self-rated health in a Norwegian population of older people participating in a preventive home visit program: a cross-sectional study

**DOI:** 10.1186/s12877-021-02269-9

**Published:** 2021-06-05

**Authors:** Astrid Fjell, Berit Cronfalk Seiger, Monica Hermann, Arvid Rongve, Jörg Aßmus, Lars Kvinge, Åke Seiger, Knut Skaug, Anne-Marie Boström

**Affiliations:** 1 Department of Neurobiology, Care Sciences and Society, Division of Nursing, Karolinska Institutet, Stockholm, Sweden; 2 Department of Health and Caring Sciences, Western Norway University of Applied Sciences, Bjørnsonsgate 45, 5528 Haugesund, Norway; 3 Department of Nursing Science, Ersta Sköndal Bräcke University College, Huddinge, Sweden; 4 Department of health and caring sciences, Western Norway University of Applied Sciences, Stord, Norway; 5 Department of Research and Innovation, Helse Fonna, Haugesund Hospital, Haugesund, Norway; 6 Inst. of Clinical Medicine, University of Bergen, Bergen, Norway; 7 Centre for Clinical Research, Haukeland University Hospital, Bergen, Norway; 8 Department of Neurobiology, Care Sciences and Society, Division of Clinical geriatrics, Karolinska Institutet, Stockholm, Sweden; 9 Department of Research and Innovation, Helse Fonna HF, Haugesund, Norway; 10 Theme Aging, Karolinska University Hospital, Huddinge, Sweden

**Correction to: BMC Geriatr 20, 323 (2020)**

**https://doi.org/10.1186/s12877-020-01733-2**

Following publication of the original article [1], the authors identified some errors in one of the authors’ names as well as incorrect date in Table 4. Correct author name and Table 4 data should be as per below:

Berit (B. first name) Cronfalk Seiger (last name).

Table 4 data - Hearing − 0.03 (0.11,0.18) and Life orientation: − 0.18 (0.04, 0.39).
